# Transcriptome profiling revealed salt stress-responsive genes in *Lilium pumilum* bulbs

**DOI:** 10.3389/fpls.2022.1054064

**Published:** 2022-11-09

**Authors:** Kyongsok So, Unil Pak, Shaoying Sun, Yiping Wang, Hao Yan, Yanni Zhang

**Affiliations:** ^1^ College of Landscape Architecture, Northeast Forestry University, Harbin, China; ^2^ Laboratory for Landscape Architecture, Institute of Architectural Material, State Academy of Sciences, Pyongyang, Democratic People’s Republic of Korea; ^3^ Department of Biotechnology, Faculty of Life Science, Pyongyang University of Science and Technology, Pyongyang, Democratic People’s Republic of Korea

**Keywords:** *Lilium pumilum*, salt stress, bulb organ, transcriptome analysis, ornamental plant

## Abstract

*Lilium pumilum* is an important ornamental, culinary and medicinal bulbous plants with salt tolerance. However, salt tolerance of lily, particularly the bulb, has been studied relatively little, which brings challenges to the cultivation of lily varieties with high salt tolerance. Here, we performed transcriptome sequencing on the bulb organs of *L. pumilum* under salt stress treatment, analyzed differential gene expressed levels and then identified several key genes associated with salt stress tolerance at genome-wide scale. For the first time, we revealed the obvious response against salt stress for *L. pumilum* bulb organs, while distinct from those for root organs. Several key genes obtained through transcriptome analysis and DEG screening include NF-YB3 transcription factor, metallothionein type 2 protein, vicilin like seed storage protein and bidirectional sugar transporter SWEET14. Rather than typical ROS scavengers like superoxide dismutase, peroxidase, and glutathione transferase, non-typical ROS scavengers such as the metallothionein type 2 protein, and vicilin like seed storage protein were upregulated in our work. The bidirectional sugar transporter SWEET14 protein and the hormone signaling proteins such as E3-ubiquitin protein ligases, PYL4 and protein phosphatase 2C were also upregulated, suggesting the role of sugars and hormones in the bulb organ responses to salt stress. Co-expression analysis of the DEGs further confirmed that NF-YB3 transcription factor acted as a hub gene, suggesting that salt stress can promote flowering of *L. pumilum*. Taken together, we identified important candidate genes associated with salt tolerance of the *L. pumilum* bulb organs, which may provide the excellent basis for further in-depth salt tolerance mechanisms of the lily bulbs.

## Introduction

Lilium, a genus of herbaceous flowering plant growing from bulbs, is widely cultivated horticultural crop. The flowers of these plants are popular in ornamental gardens or indoor potted plants. Lilies are also used in the culinary industry and folkloric medicine (Zhou et al., 2021). *Lilium pumilum*, commonly known as coral lily, is native to grassy mountainous areas of northeast China which are heavily salinized region of the country. Therefore, *L. pumilum* is regarded as a salt tolerant plant, but the molecular mechanism of salt tolerance in *L. pumilum* has not been extensively studied, which limits its widespread application.

Salt stress is a major abiotic stress which adversely impacts plant growth and development (Shrivastava and Kumar, 2015). Worldwide distribution of saline-alkali land and soil salinization of non-saline land have been significantly reducing the cultivable land areas, challenging the agricultural, horticultural and forestry production. Salt accumulation in arable soils is caused by seawater and irrigation water that contains trace amounts of sodium chloride (NaCl) (Flowers and Yeo, 1995; Tester and Davenport, 2003). Damages caused by salt stress are classified into two categories of osmotic stress and ionic toxicity and several salt tolerant plants including haplotypes have evolved salt tolerance responses as follows: In early salt stress period, osmotic homeostasis was biased by accumulated salts in soil, decreasing the water absorbing ability of plants and accumulation of osmolytes such as sucrose is one of the natural responses of plants to recover the osmotic homeostasis, ultimately lowering water loss and maximizing water uptake. Later, salt ions accumulate within plant cells and cause ion toxicity, affecting intracellular metabolic processes and Na^+^ exclusion and vacuole compartmentalization are another typical plant responses to minimize the ion toxicity (Blumwald, 2000; Sahi et al., 2006; Munns and Tester, 2008; Flowers and Colmer, 2015). In previous studies, multiple factors have been found to play crucial roles in the above salt stress responses in plants (Zhao et al., 2021); which include histidine kinase receptor protein HK1, Ca^2+^ dependent signaling factors like calcium-dependent protein kinases (CDPKs), calcineurin B-like proteins (CBLs) with CBL-interacting protein kinases (CIPKs) and calmodulin-binding transcription activators (CAMTAs), transcription factors such as basic leucine zipper (bZIP), WRKY, APETALA2/ETHYLENE RESPONSE FACTOR (AP2/ERF), MYB, basic helix–loop–helix (bHLH) and NAC families, Na^+^ and K^+^ transporters like CYCLIC NUCLEOTIDE-GATED CHANNEL (CNGC) and the GLUTAMATE-LIKE RECEPTOR (GLR) families, tonoplast-localized Na^+^/H^+^ exchanger 1 (NHX1), plasma membrane-localized SALT OVERLY SENSITIVE 1 (SOS1) Na^+^/H^+^ antiporters, high-affinity K^+^ transporter HKT, ROS scavenging enzymes like superoxide dismutase, peroxidase, and glutathione transferase, organic osmolytes like proline, glycine betaine, sugar alcohols, polyamines, and proteins from the late embryogenesis abundant (LEA) superfamily (Deinlein et al., 2014). Phytohormones such as abscisic acid (ABA), auxin, cytokinin (CK), gibberellic acid (GA), brassinosteroids (BRs), ethylene, jasmonic acid (JA), salicyclic acid (SA) and strigolactones (SLs) are also important factors that regulate plant responses to salt stress (Zhao et al., 2021). These hormones are classified into two groups as stress hormones such as ABA, SA, JA, ethylene and growth hormones including auxin, CKs, and act in a sophisticated crosstalk (Yu et al., 2020). In addition, the mitogen activated protein kinase (MAPK) cascade has been ubiquitously found in salt stress responses of many plant species, and it is believed to function as point of convergence in plant development and signaling associated with hormones and stresses (Sinha et al., 2014).

The advent of ‘Omics’ technologies such as the transcriptome analysis have been rapidly developed and widely applied to plants, enabling the genome-wide identification of key regulatory factors in plant responses to various stresses (Imadi et al., 2015; Zhu et al., 2015; Ju et al., 2021). Transcriptome analysis *via* RNA-sequencing (RNA-seq) can provide dynamic nature of transcriptome at higher coverage and greater resolution than previously employed methods such as Sanger sequencing- and microarray (Kukurba and Montgomery, 2015). RNA-seq has already been successfully applied to *L. pumilum* and revealed key genes associated with dormancy release at cold storage condition (Wang et al., 2018) and tepal trichome development (Xin et al., 2021). The response of *L. pumilum* bulbs to salt stress has not yet been studied using RNA-seq. In recent years, it has been reported that transcription factors from *L. pumilum* can improve plant salt tolerance (Yong et al., 2019; Wang et al., 2020; Wang et al., 2022; Yan et al., 2022). But they have not yet been studied at genome-wide scale using RNA-seq. Root is the very organ that first senses and responds to salt stress and leaf is the organ that first shows apparent morphological changes upon salt stress. Therefore, most of studies on salt stress have focused on the root and leaf. Bulbous plants such as *L. pumilum* have unique organs called the bulb which is known to function as nutrient storage or flowering organs. It is believed that *L. pumilum* is salt-tolerant ornamental plant which may be related to its unique bulb organ function. But it requires evidences showing that the bulb organs help the plants to overcome stressful environments such as salt stress. Therefore, RNA-seq analysis of lily bulb organs can provide novel insights into the salt stress tolerance of *L. pumilum*.

In this study, we analyzed the transcriptome data of the *L. pumilum* bulb organs treated with salt stress in order to identify key genes associated with salt stress responses of bulb organs at genome-wide scale. The transcriptome data of *L. pumilum* bulb organs were generated by Illumina sequencing and Trinity *de novo* assembly. The genes showing differential expression (DEGs) in the *L. pumilum* bulb organs upon salt stress treatment were then identified by comparing the gene transcript profiles in the bulb organ samples between salt-treated and control plants at different time points of salt stress treatment, mainly focusing on identifying the candidate genes associated with transcription regulation, ROS scavenging, ion detoxification, osmolyte accumulation and hormone signaling. Putative hub genes were predicted through building co-expression network using STRING database and Cytoscape software. The expression levels of several key genes were validated by qRT-PCR. The preliminary results of this study provided the basic information on molecular mechanism underlying regulatory network for salt stress responses in the *L. pumilum* bulb organs that may serve as foundation for further in-depth functional studies.

## Materials and methods

### Plant materials and salt stress treatment

*L. pumilum* plants were grown in laboratory at College of Landscape Architecture of Northeast Forestry University (Harbin, Heilongjiang, China). Healthy seedlings were cultured on hydroponic tank supplemented with Hoagland nutrient solution at 70% relative humidity, and 22-25 °C ambient temperature. 3 wk-old plants were treated with 150 mM NaCl for 12 h. The plants at 0 h of NaCl treatment were used as control. Bulb organ samples were collected at 2 h and 12 h, respectively.

### RNA extraction, cDNA library construction, transcriptome sequencing and *de novo* assembly

Total RNA was extracted from the bulb organ samples of different sampling times using Trizole reagent (TG-DP441, China). The quantity and purity of the extracted RNA samples were evaluated by Bioanalyzer 2100 and RNA 100 Nano LabChip Kit (Agilent, CA, USA) with RIN number >7.0. PolyA RNA was obtained by purification of total RNA with oligo-dT magnetic beads. Then, mRNA was treated with divalent cations under temperature gradient for fragmentation into smaller size. Next, cDNA library was constructed by reverse-transcription reactions from those cleaved RNA fragments using RNA-seq sample preparation kit (Illumina, San Diego, USA). Finally, the cDNA library was sequenced on Illumina Novaseq™ 6000 platform (LC Sciences, USA). All raw reads were deposited in the Sequence Read Archive (SRA) database in NCBI with accession number PRJNA851552. Clean reads were obtained by removing adaptor contamination, low quality bases as well as undetermined bases using Cutadapt (Martin, 2011) and in-house Perl scripts and their quality was then assessed by FastQC (http://www.bioinformatics.babraham.ac.uk/projects/fastqc/). Trinity 2.4.0 (Grabherr et al., 2011) was utilized to assemble the clean data. The *de novo* assembled transcripts were clustered and then the overlapping reads were counted to obtain unigenes for further analysis. All unigenes obtained by *de novo* assembly were blasted to NCBI non-redundant protein database (NR), Gene ontology (GO), Kyoto Encyclopedia of Genes and Genomes (KEGG), Pfam, swiss-Prot, and eggNOG database using DIAMOND (Buchfink et al., 2015) with threshold of e-value <0.00001.

### Differential expression analysis

Expression levels of the unigenes were determined by calculating TPM (Transcripts Per Kilobase of exon model per Million mapped reads) (Mortazavi et al., 2008). DEGs were screened out according to the criteria of log2 (fold change) >1 or <-1 with p value <0.05 (statistically significant) using R package-edgeR (Robinson et al., 2009). The DEGs were further annotated to GO and KEGG databases for their functional enrichment analysis. In GO enrichment analysis, all the DEGs were mapped to corresponding GO terms, the gene number in each term was calculated and then the GO terms significantly enriched in DEGs were found through the hypergeometric test. KEGG enrichment analysis for the DEGs was also conducted to find enriched pathways for the DEGs. GO and KEGG analyses data were converted into scatter plots using ggplot2. DEGs at different periods (0-2 h and 2-12 h) of salt stress were compared to find out the key genes which were predicted as essential regulators for salt stress responses of the bulb organs in *L.pumilum*.

### Prediction of co-expression network

In order to build co-expression network. The DEGs at 0-12 h with p <0.05 were selected and their interaction was predicted using STRING database (https://cn.string-db.org/cgi/input?sessionId=bOrBIG9AvMjL&input_page_show_search=on ). The *Arabidopsis thaliana* was used as reference organism in the prediction. Then, the network was built using Cytoscape. Network analysis and hub gene identification were conducted using network analyzer and cytohubba plugins, respectively, in Cytoscape.

### Quantitative real-time reverse transcription-PCR

Several key DEGs were randomly selected for qRT-PCR to verify their expression levels. RNA was extracted and then reversely transcribed into cDNA using Rever Tra Ace qPCR RT Kit (TOYOBO, Osaka, Japan) by a reaction flow of 37 °C, 15 min and 98 °C, 5 min. Primers were designed using the RealTimePCR design tool of the IDT website (http://sg.idtdna.com/scitools/Applications/RealTimePCR ) and were synthesized by Comate Bioscience (Jilin, China). Primer information was shown in Additional file. qRT-PCR was performed using triazole reagent UltraSYBR Mixture kit (CWBIO, Beijing, China). The 20 μl reaction system was as follows (**Table 1**):

**Table 1:** qRT-PCR reaction system.

Reagent	Volume (μl)
2x UltraSYBR Mixture	10
Upstream primer	0.4
Downstream primer	0.4
template	0.8
ddH_2_O	8.4

qRT-PCR reactions were performed in Roche Light Cycler 96 (Roche, Basel, CH) according to following program: preincubation, 95 °C for 10 min; three steps amplification, 95 °C for 10 s, 60 °C for 30 s, and 72 °C for 32 s (total 40 cycles); melting, 95 °C for 15 s, 60 °C for 1 min, 95 °C for 15 s; cooling, 37 °C for 30 s. Lily *Actin* was used as an internal reference gene. All experiments were performed in three biological replicates. Relative expression level was calculated by the 2^-ΔΔCt^ method.

## Results

### Transcriptome sequencing and assembly

Total RNA of the bulb samples of *L. pumilum* were sequenced by Illumina double-terminal sequencing technology. Three biological replicates, a total of 9 samples, were generated for each time point. The sequencing data of all samples exceeded 6 G. In total, 492572310 raw sequencing reads were obtained from the control and treated samples and were then subjected to cleaning overrepresented sequences such as Illumina adaptor sequence, generating 455124918 clean reads that were used for subsequent analyses (**Table 2**).

**Table 2:** Summary of the sequences analyzed for *L. pumilum* bulb samples.

Sample	Raw_Reads	Valid_Reads	Valid_Bases	Valid%	Q20%	Q30%	GC%
C_bulb1	57258018	55127478	7.73G	96.28	98.69	95.47	49.60
C_bulb2	58619530	56504610	7.92G	96.39	98.64	95.36	49.73
C_bulb3	57154732	55385518	7.76G	96.90	98.62	95.27	49.64
N_bulb11	50511204	44159970	6.18G	87.43	98.58	95.36	50.00
N_bulb12	54081656	45886318	6.43G	84.85	98.69	95.64	50.00
N_bulb13	54751366	45860366	6.43G	83.76	98.72	95.69	50.03
N_bulb21	50637620	48891748	6.85G	96.55	98.63	95.32	49.76
N_bulb22	55207422	50204940	7.04G	90.94	98.75	95.72	50.58
N_bulb23	54350762	53103970	7.45G	97.71	98.71	95.54	49.90
Summary	492572310	455124918	63.79G				

(The numbers 1-3 after C_bulb, N_bulb1 and N_bulb2 represent the three independent biological replicates for the control (0 h), 2 h and 12 h duration of salt stress treatment, respectively.

Q20: The percentage of bases with a Phred value > 20.

Q30: The percentage of bases with a Phred value > 30).

**Figure 1:**
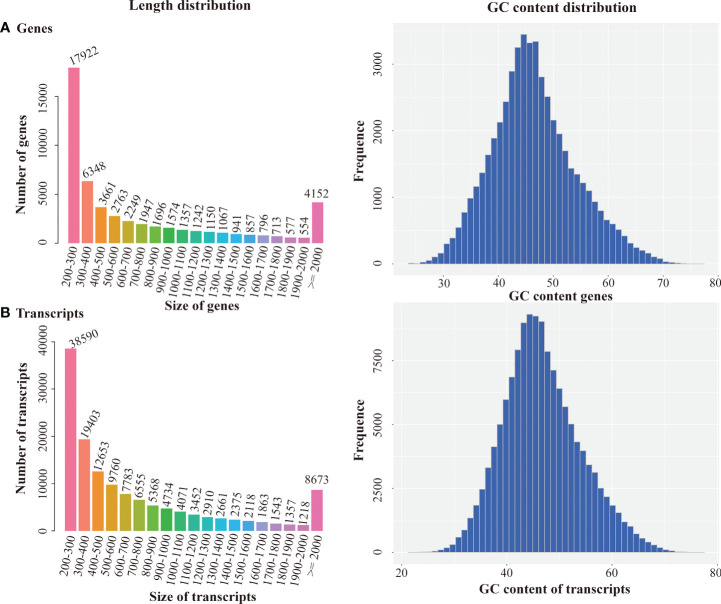
Length distribution and GC content distribution of Genes **(A)** and Transcripts **(B)**.

Q20, Q30, and GC content were over 98, 95, and 49%, respectively, indicating high quality of sequencing results. The sequencing data were assembled by Trinity. 137087 transcripts and 51566 unigenes were obtained, with an average length of 481 bp and 436 bp, respectively (**Table 3**). Length distribution and GC content of the transcripts and unigenes represented high quality of assembly works (**Figure 1**). The N50 value for transcript and unigene was 1132 and 1331, respectively, suggesting that the assembled data were competent for subsequent analyses.

**Table 3:** Summary of trinity assembly result.

Index	All	GC%	Min Length	Medium Length	Max Length	Total Assembled Bases	N50
Transcript	137087	46.38	201	481	16120	103421503	1132
Gene	51566	46.49	201	436	16120	40504035	1331

### Functional annotation of all non-redundant unigenes

*De novo* assembled sequencing data were annotated to six databases namely NR, GO, KEGG, Pfam, swiss-Prot, and eggNOG using the DIAMOND program. The annotated number of unigenes for each database is shown in **Table 4**.

**Table 4:** Annotated number and ratio of the unigenes in various databases.

Databases	Number of unigenes	Ratio (%)
All	51566	100.00
NR	26510	51.41
GO	22213	43.08
KEGG	17821	34.56
Pfam	20430	39.62
swissprot	18678	36.22
eggNOG	25374	49.21

**Figure 2:**
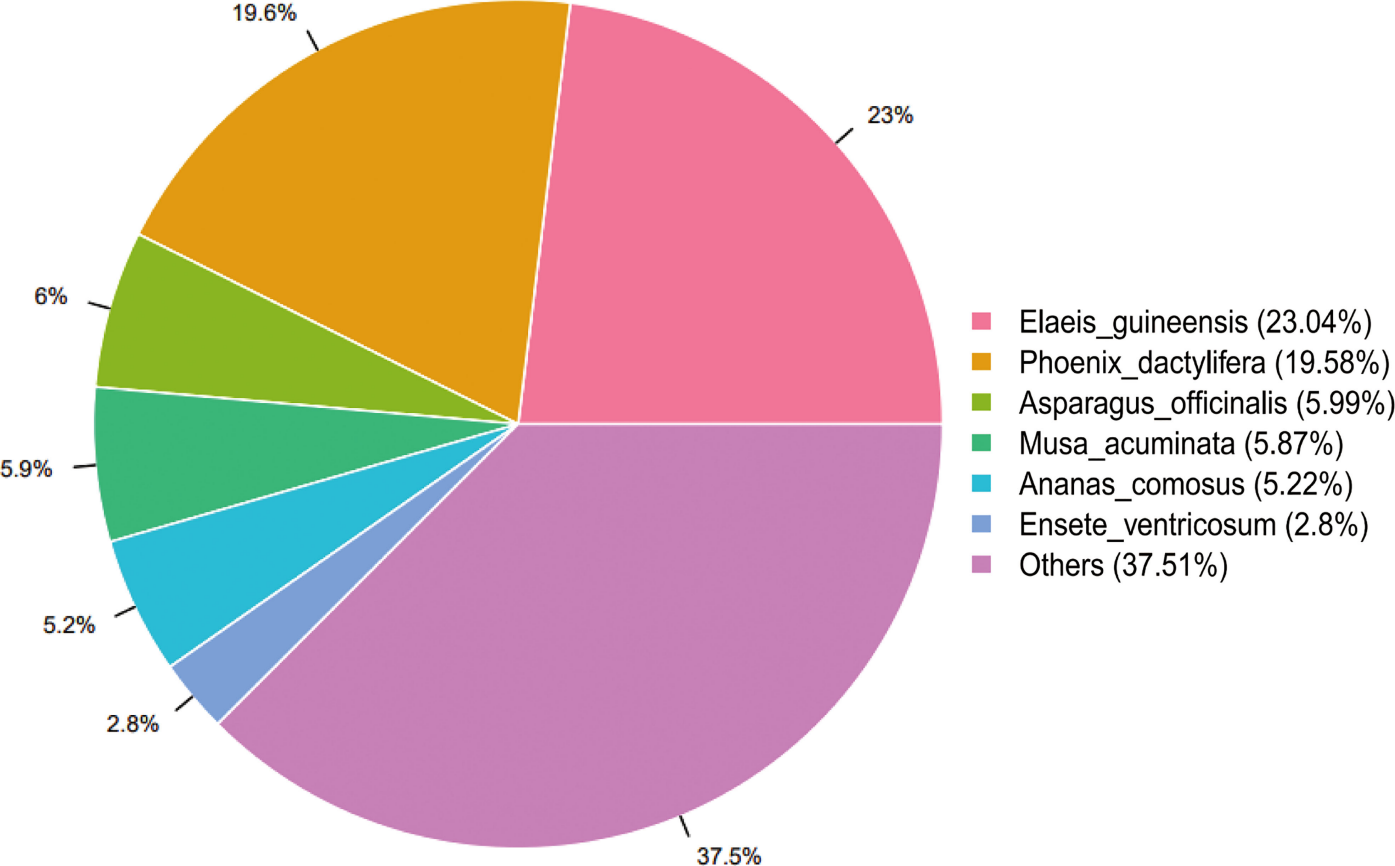
Species distribution for the assembled unigenes annotated to NCBI non-redundant protein database (NR). The percentages represent genetic homology degree of assembled unigenes with respective species.

All unigenes were annotated at least by one database. Most unigenes (26510; 51.41%) were annotated to NR. According to the annotation result to NR database, the unigenes of *L. pumilum* bulb showed high homology with *Elaeis guineensis* (23.04%) and *Phoenix dactylifera* (19.58%) (**Figure 2**).

**Figure 3:**
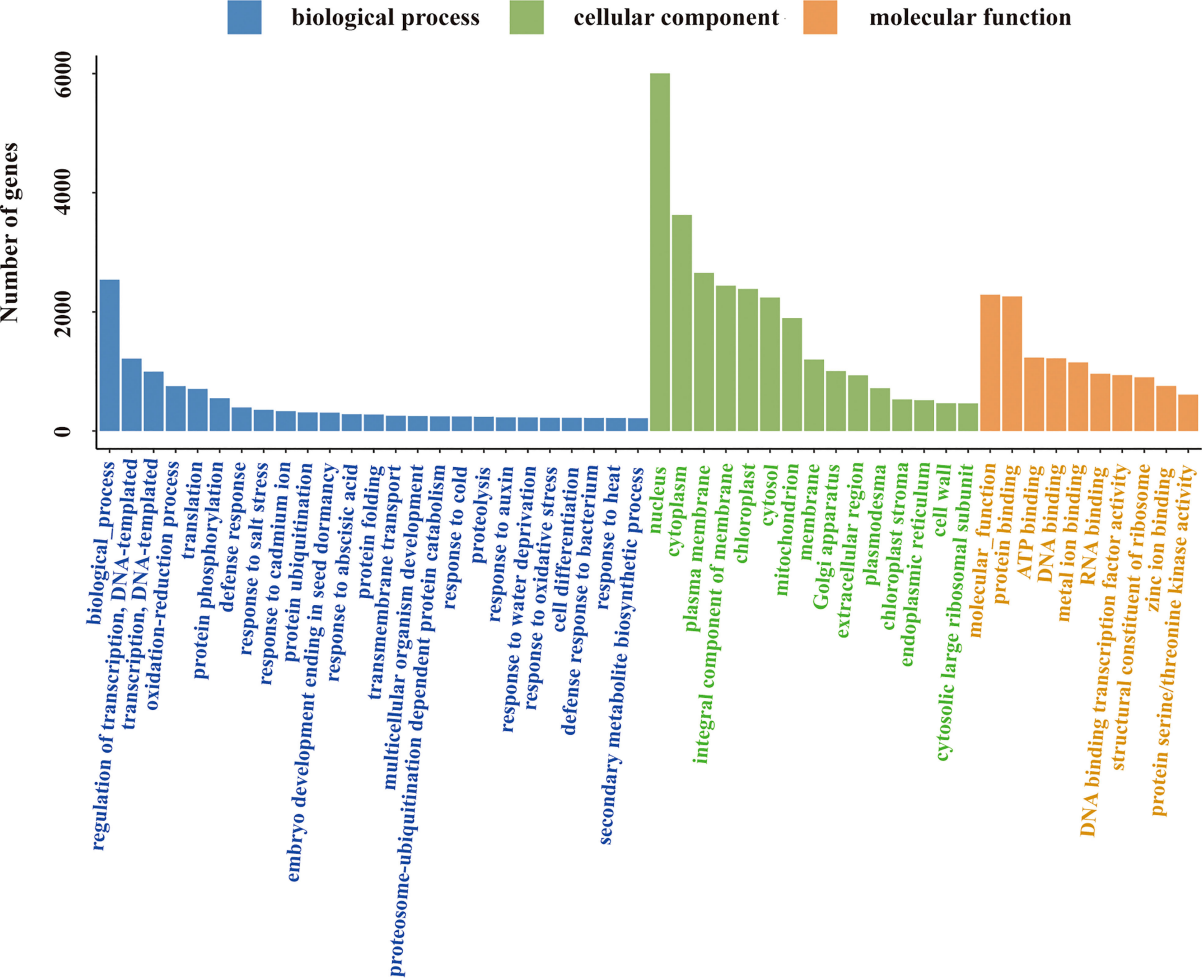
Classification of the unigenes.

**Figure 4:**
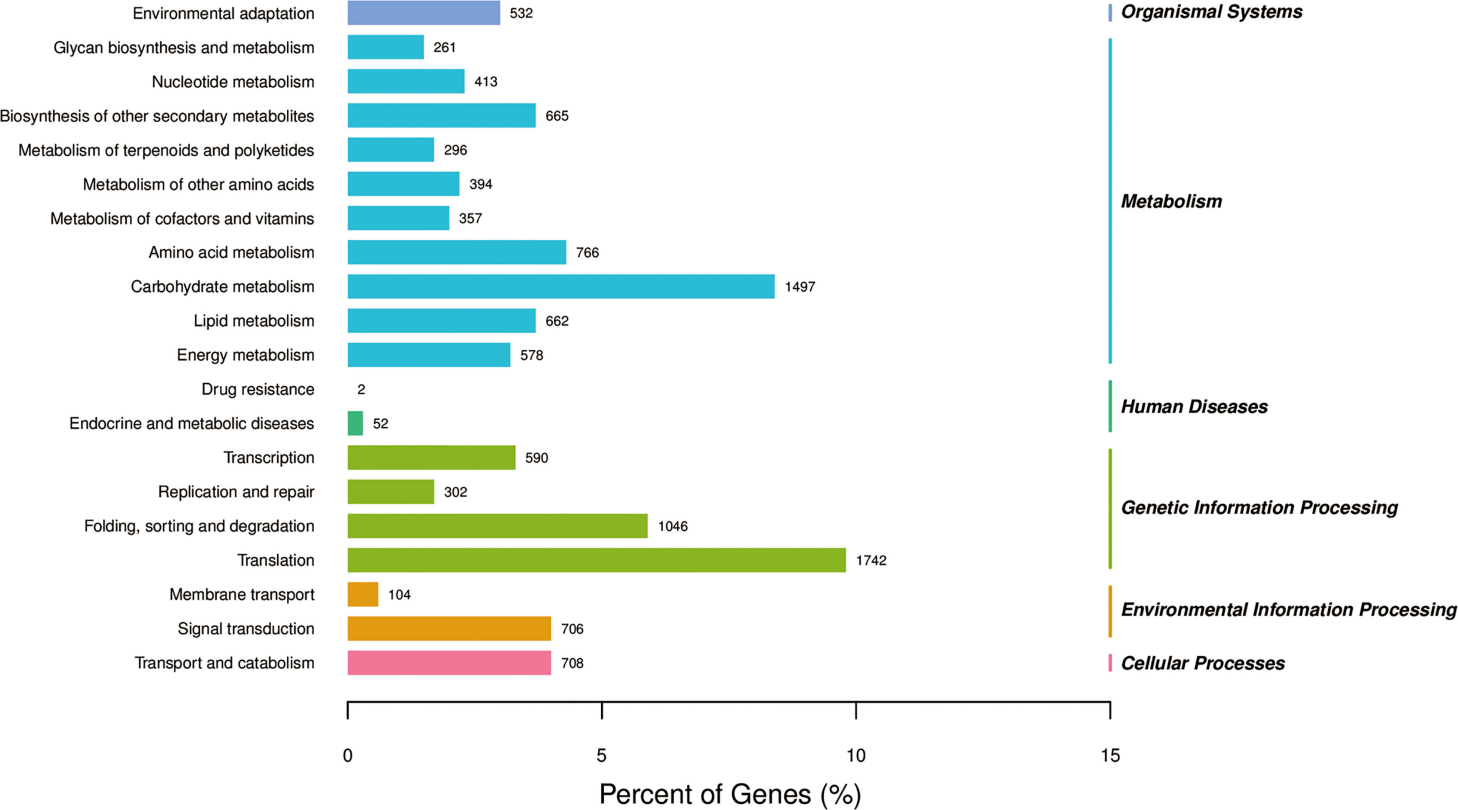
KEGG pathway categorization of the unigenes.

**Figure 5:**
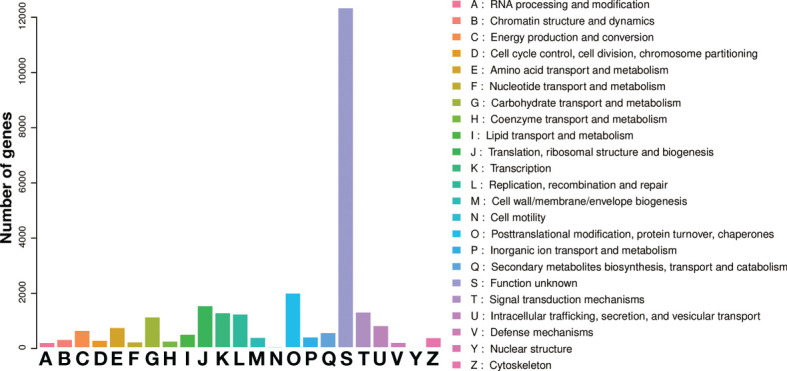
eggNOG classification of the unigenes.

All annotated unigenes were functionally classified by GO, KEGG, and eggNOG databases. In the GO ontology annotation, unigenes were grouped into 3 categories of “biological process”, “cellular component”, and “molecular function” (**Figure 3**). In the category of “biological process”, several activities including gene transcription and translation, protein modification and degradation, secondary metabolism, and responses to salt, cold, heat, water and oxidative stresses as well as hormones like auxin and abscisic acid were found in salt stress response of *L. pumilum*. In the categories of “cellular component” and “molecular function”, various cellular components and molecular functions such as nucleus, cytoplasm, membranes, chloroplast, and mitochondria and protein binding, DNA binding, metal ion binding, etc were enriched in salt stress response of *L. pumilum*. In the KEGG annotation, gene transcription and translation also showed the highest activities and environmental adaptation and secondary metabolilsm were the most active metabolism except for the housekeeping metabolisms such as carbohydrate, lipid, amino acid metabolisms (**Figure 4**). Annotation by the eggNOG database also revealed similar patterns in classification of the unigenes (**Figure 5**). Enrichment analysis of DEGs among the unigenes annotated by GO and KEGG will be analyzed in the next section.

### Identification of DEGs related to salt stress responses

**Figure 6:**
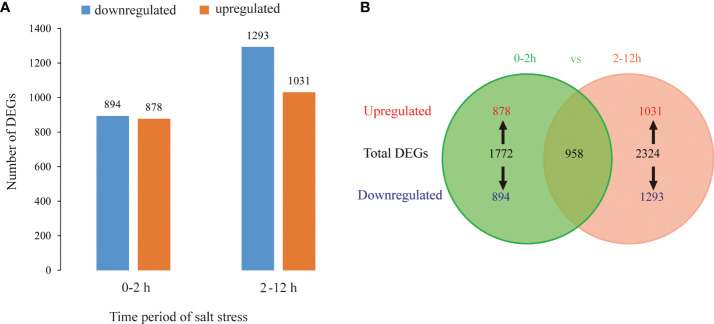
DEGs between *L. pumilum* bulb samples at different time points (0, 2, and 12 h) during salt stress. **(A)** Number of upregulated or downregulated DEGs in pairwise comparisons, **(B)** Venn diagram showing number of DEGs in different comparisons.

Among the samples (0, 2 h, and 12 h of salt treatment), total 4096 DEGs (log_2_Fold change>1 or <-1) were identified by pairwise comparison of TPM values. 1772 (878: upregulated and 894: downregulated) DEGs were identified at 0-2 h period of salt stress, and 2324 (1031 upregulated and 1293 downregulated) DEGs at 2-12 h period of salt stress (**Figure 6A**).

**Figure 7:**
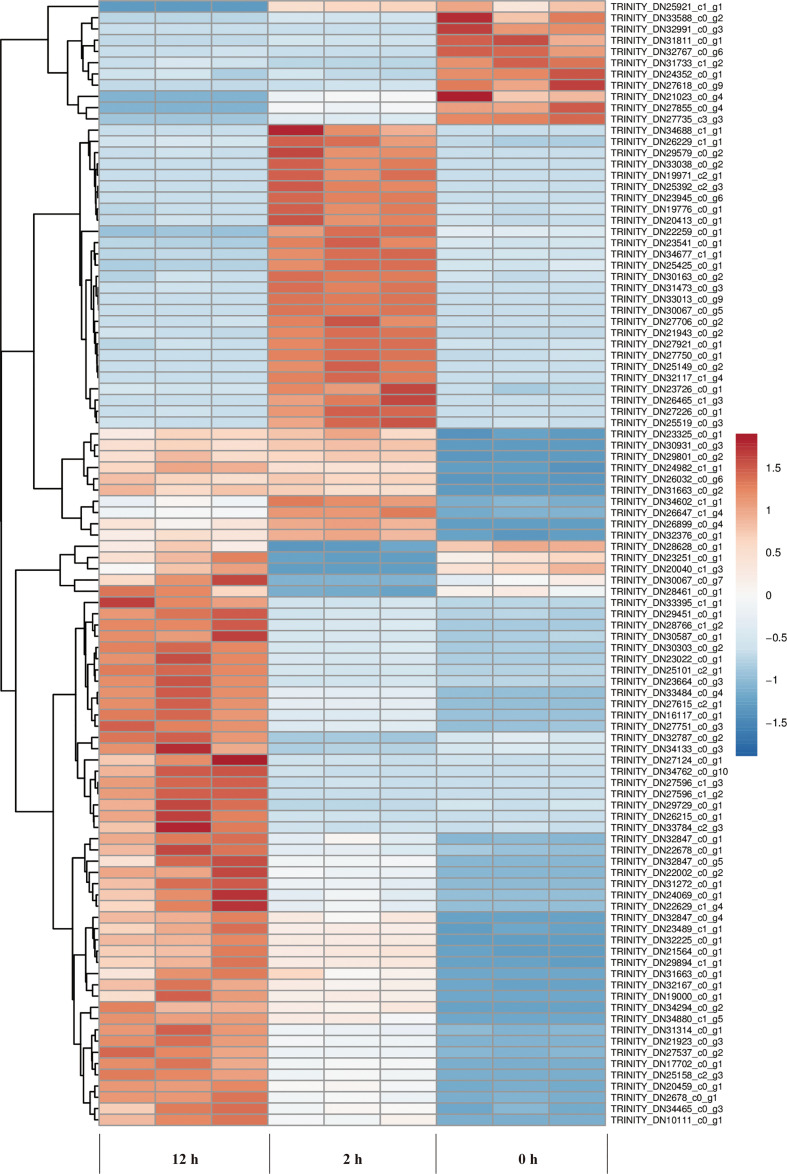
Cluster analysis of DEGs at different periods (0, 2, and 12 h) of salt stress. The abscissa represents the three independent biological replicates for the control (0 h), 2 h and 12 h duration of salt stress treatment, respectively. The ordinate represents the ID of a hundred genes with smallest p value. Color gradient from blue to red represents gene expression level from low to high.

We examined the clustering patterns of DEGs for different periods (0-2 h and 2-12 h) of salt stress by clustering the DEGs according to the similarity in their functions, metabolic processes or cellular pathways. In our study, TPM of a differential gene was displayed as Z-value, and the cluster analysis was performed using 100 DEGs with the smallest *p* value. The clustering analysis (**Figure 7**) showed that 3 biological replicates for all samples have good repeatability.

**Figure 8:**
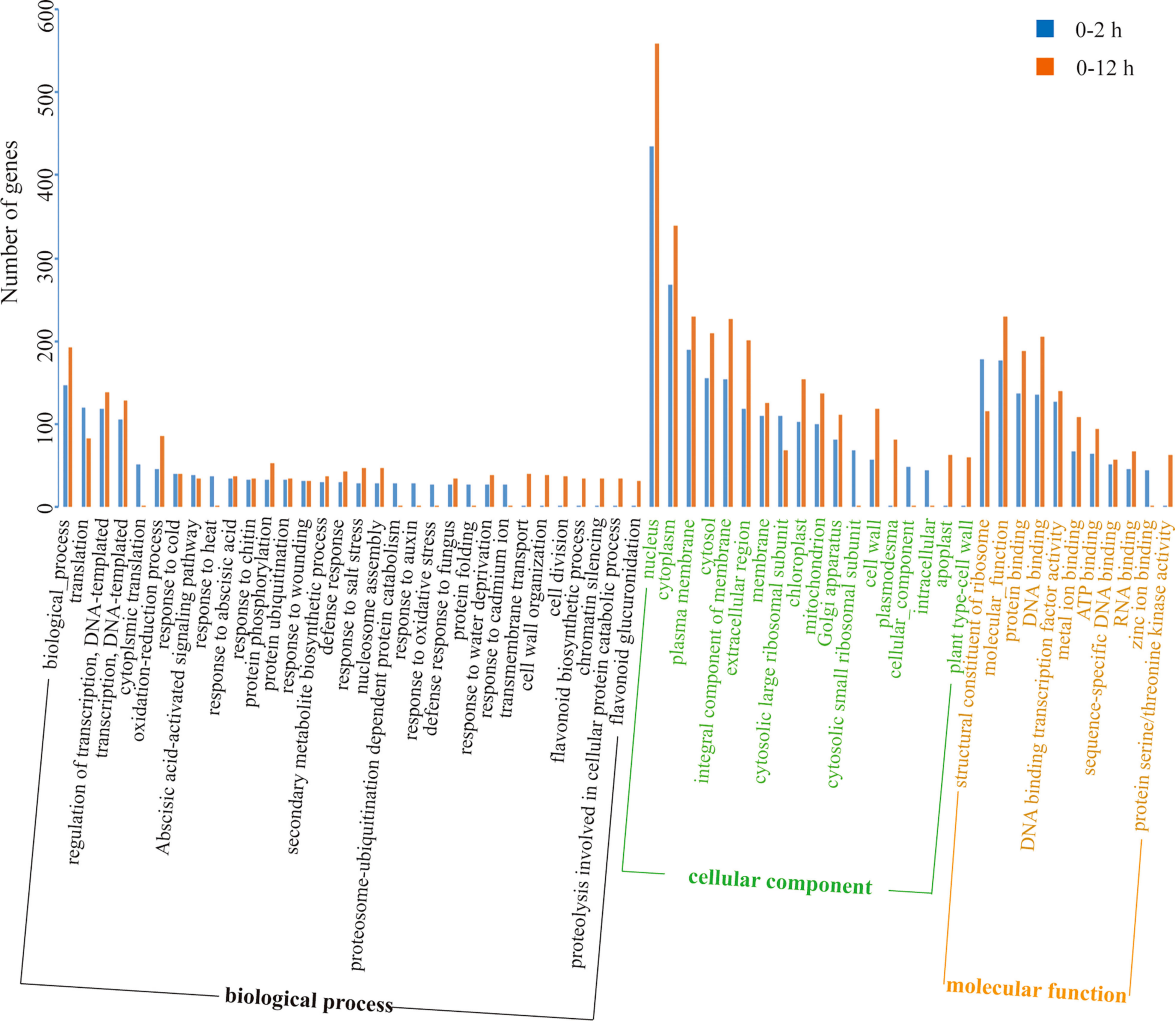
GO enrichment analysis of DEGs of two different periods (0-2 h and 2-12 h) during salt stress.

The DEGs at the initial stage (0-2 h period) and later stage (2-12 h period) during salt stress were subjected to GO enrichment analysis (**Figure 8**).

DEGs were highly enriched in the GO functions such as “biological process”, “translation”, “DNA-templated transcription and its regulation”, “oxidation-reduction processes”, “nucleus”, “cytoplasm”, “membrane”, “protein binding”, “DNA binding”, etc. The different enrichment results at the initial stage (0-2 h) and the later stage (2-12 h) indicated that different genetic programs controlled the response of lily bulb to salt stress in this study. As the salt stress prolongs, the genes that belong to the GO functions including “cytoplasmic translation”, “response to heat”, “proteosome-ubiquitination dependent protein catabolism”, “proteolysis involved in cellular protein catabolic process”, “response to auxin”, “response to oxidative stress”, “protein folding”, “response to cadmium ion”, “transmembrane transport”, “cell wall organization”, “cell division”, “chromatin silencing”, “flavonoid biosynthetic process”, “flavonoid glucuronidation”, “cytosolic small ribosomal subunit”, “plasmodesma”, “cellular component”, “intracellular”, “apoplast”, “plant type-cell wall”, “zinc ion binding”, and “protein serine/threonine kinase activity” showed dramatically differential expression. Therefore, it is obvious that the complicated responses elicited by salt stress includes a wide-range of cellular activities and the genetic program of the plant (lily bulb) has a temporal differential pattern in which the aforementioned DEGs are regulated differently depending on time.

**Figure 9:**
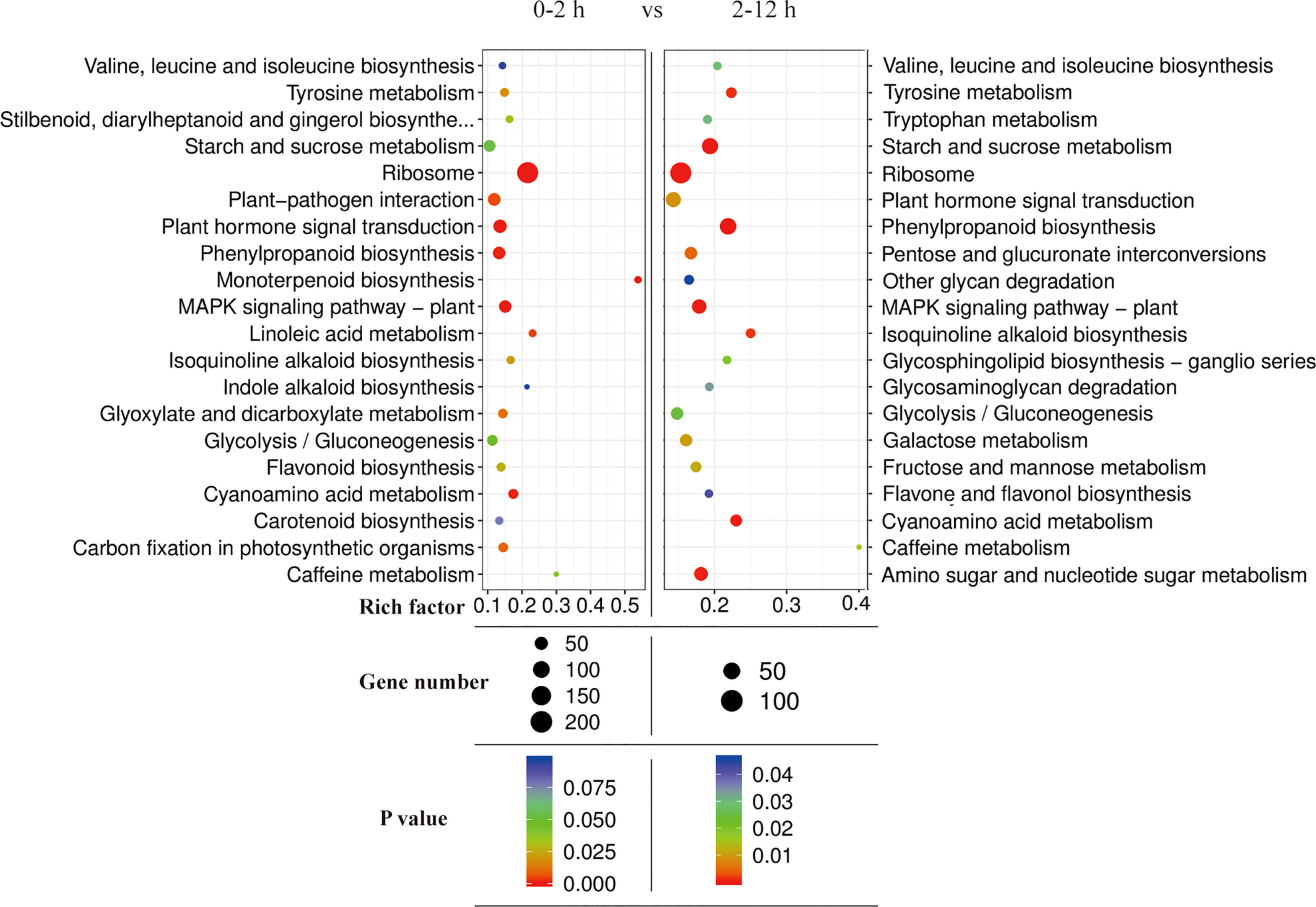
KEGG enrichment analysis of DEGs of two different periods (0-2 h and 2-12 h) during salt stress.

In addition, KEGG pathway annotation of the DEGs in two different periods (0-2 h vs 2-12 h) during salt stress showed that the DEGs were associated with more than 30 pathways and were highly enriched in the pathways associated with “Ribosomes”, “Plant hormone signal transduction”, “Phenylpropanoid biosynthesis”, “MAPK signaling pathway-plant”, and “Starch and sucrose metabolism” (**Figure 9**). DEG enrichment results at two different time periods (0-2 h and 2-12 h) also revealed the differences in pathway types and gene numbers, which might be related to temporally different responses to salt stress at 0-2 h and 2-12 h periods.

**Figure 10:**
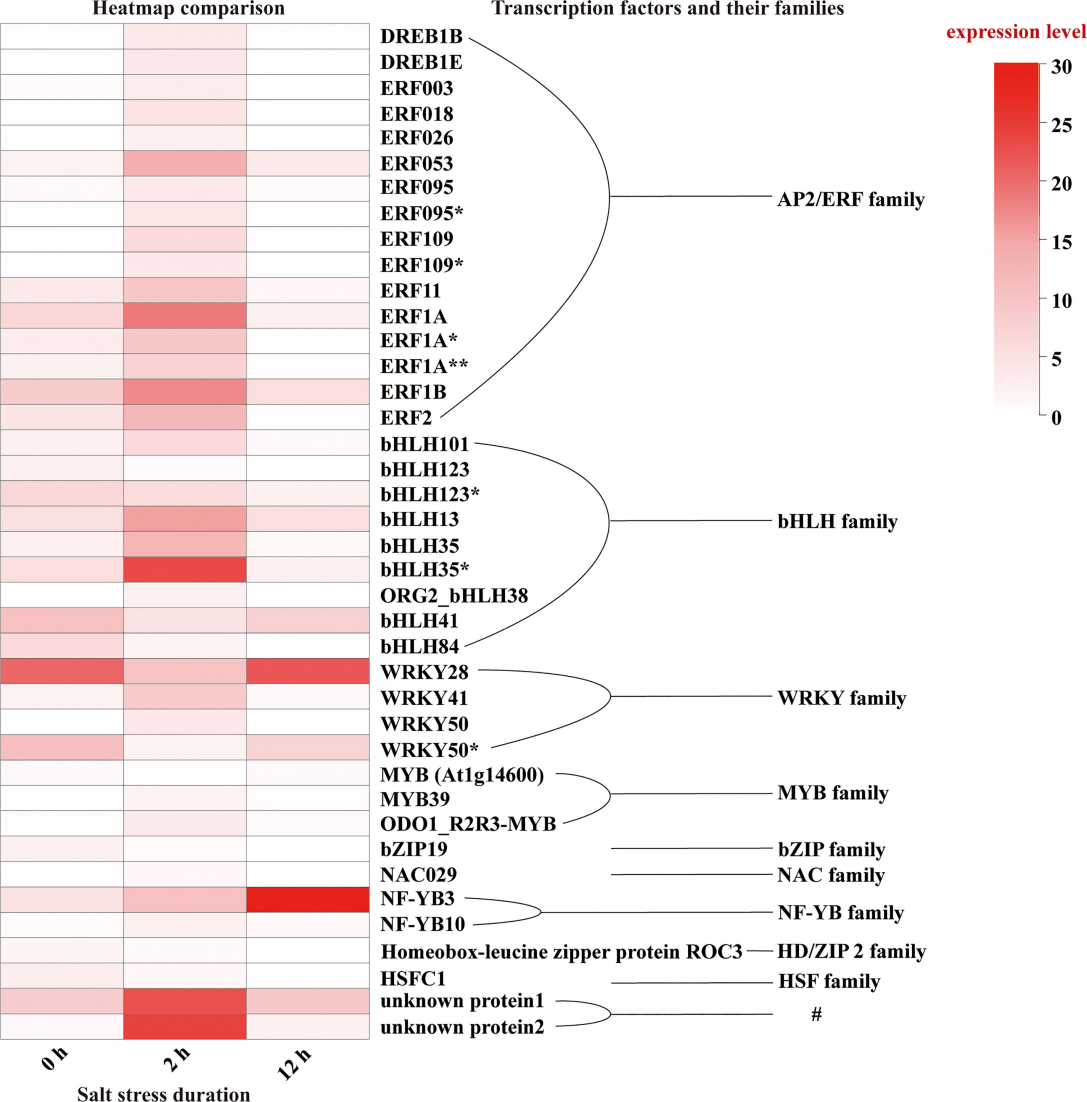
Expression pattern of the transcription factors identified in DEGs. “*” indicates the identical gene ID but different species and “#” means that they have DNA-binding transcription factor activity although they were unknown proteins.

Then, we focused on the 958 DEGs which were co-induced throughout the entire period of salt stress (0-12 h) (**Figure 6B**). Among them, we identified various transcription factors belonging to AP2/ERF, bHLH, bZIP, HD2/ZIP2, HSF, MYB, NAC, NF-YB, and WRKY families (**Figure 10**), among which AP2/ERF, bHLH, WRKY transcription factors take the highest proportion. A few unknown proteins with DNA-binding transcription factor activity also showed remarkably significant differential expression patterns during salt stress. Of all, NF-YB3 transcription factor showed high and constant upregulation during the throughout entire salt stress period (0-12 h). Besides, bHLH35*, bHLH13, ERF053, ERF1A, ERF1B, and WRKY28 showed remarkable upregulation albeit not constant for entire period. bHLH35*, bHLH13, ERF053, ERF1A, and ERF1B were highly upregulated at the initial period (0-2 h) during salt stress treatment and then declined, indicating their roles in Lily bulb’s primary responses to salt stress. In contrast, WRKY28, WRKY50*, and bHLH41 were downregulated during the initial period, and then upregulated later, suggesting that they might have distinguishing roles other than bHLH35*, bHLH13, ERF053, ERF1A, and ERF1B.

**Figure 11:**
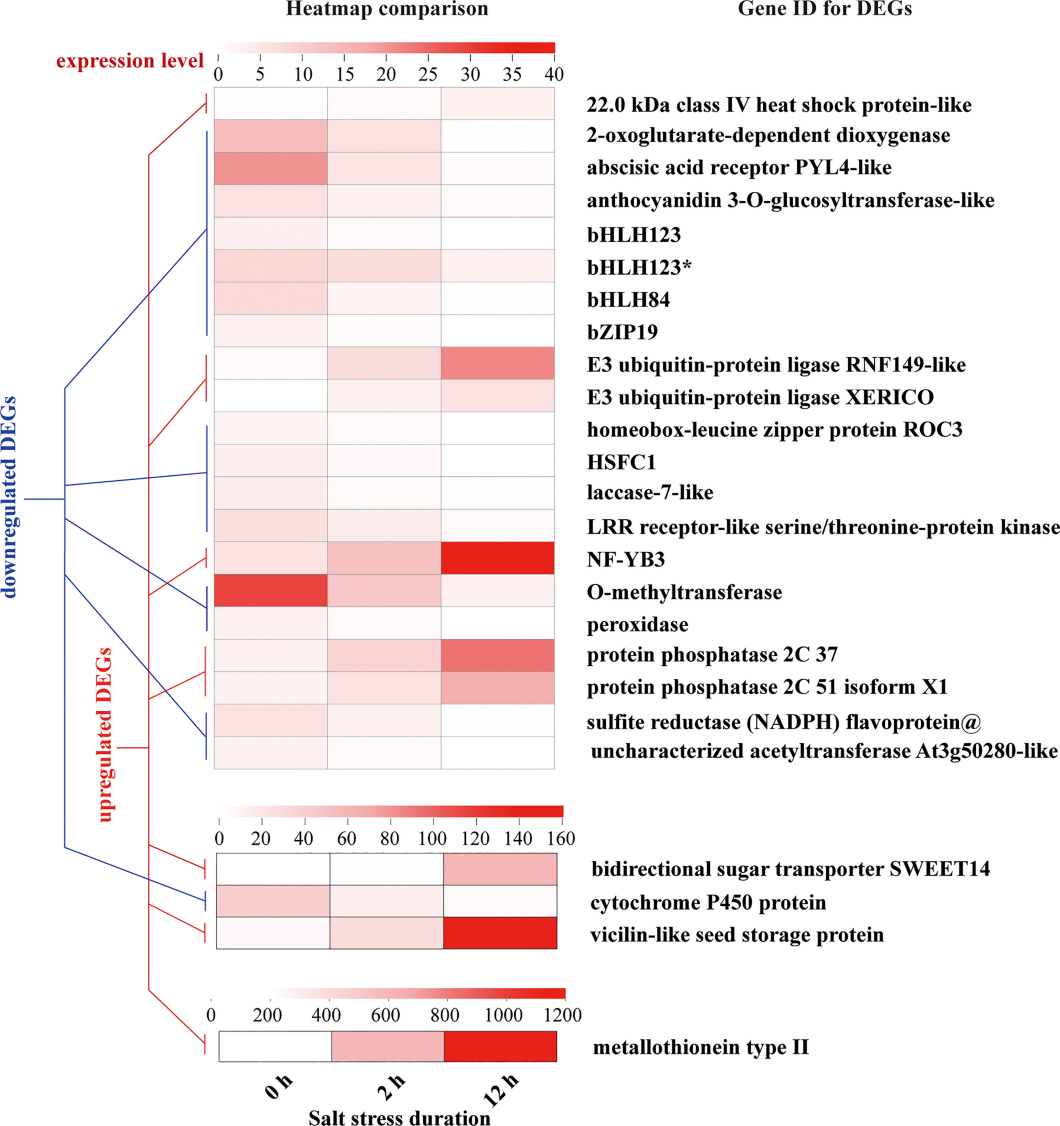
DEGs with the tendency of constant upregulation or downregulation during entire period (0-12 h) of salt stress. Each Gene ID of DEGs represents putative or predicted name based on one discovered in other eukaryotic organisms. “*” indicates the identical gene ID but different species.

In addition, 48 of the 958 DEGs showed the continuous upregulation or downregulation during entire period (0-12 h), and only 25 genes with annotated information showed temporal differential expression (**Figure 11**).

We classified these genes into 6 categories: stress responses (heat shock protein, 2-oxoglutarate-dependent dioxygenase, laccase, LRR receptor-like serine/threonine protein kinase, O-methyltransferase, peroxidase, cytochrome P450 protein, metallothionein type 2), hormone signaling (abscisic acid receptor PYL, E3 ubiquitin-protein ligases, protein phosphatases), primary metabolism for energy conservation (sulfite reductase flavoprotein), secondary metabolite production (anthocyanidin 3-O-glucosyltransferase, O-methyltransferase, acetyltransferase, bidirectional sugar transporter SWEET14), seed development (vicilin like seed storage protein), and transcription factors (bHLH123, bHLH84, bZIP19, homeobox-leucine zipper protein ROC3, HSFC1, NF-YB3). In the whole process of salt stress, the expression of bHLH84, bHLH123, bHLH123*, bZIP19, homeobox-leucine zipper protein ROC3, HSFC1 was downregulated, and the expression of NF-YB3 was upregulated 3-6 times. Among other DEGs, metallothionein type 2 protein showed unusually high expression levels and strong upregulation in response to salt stress treatment.

### Prediction of co-expression network and identification of putative hub genes

**Figure 12:**
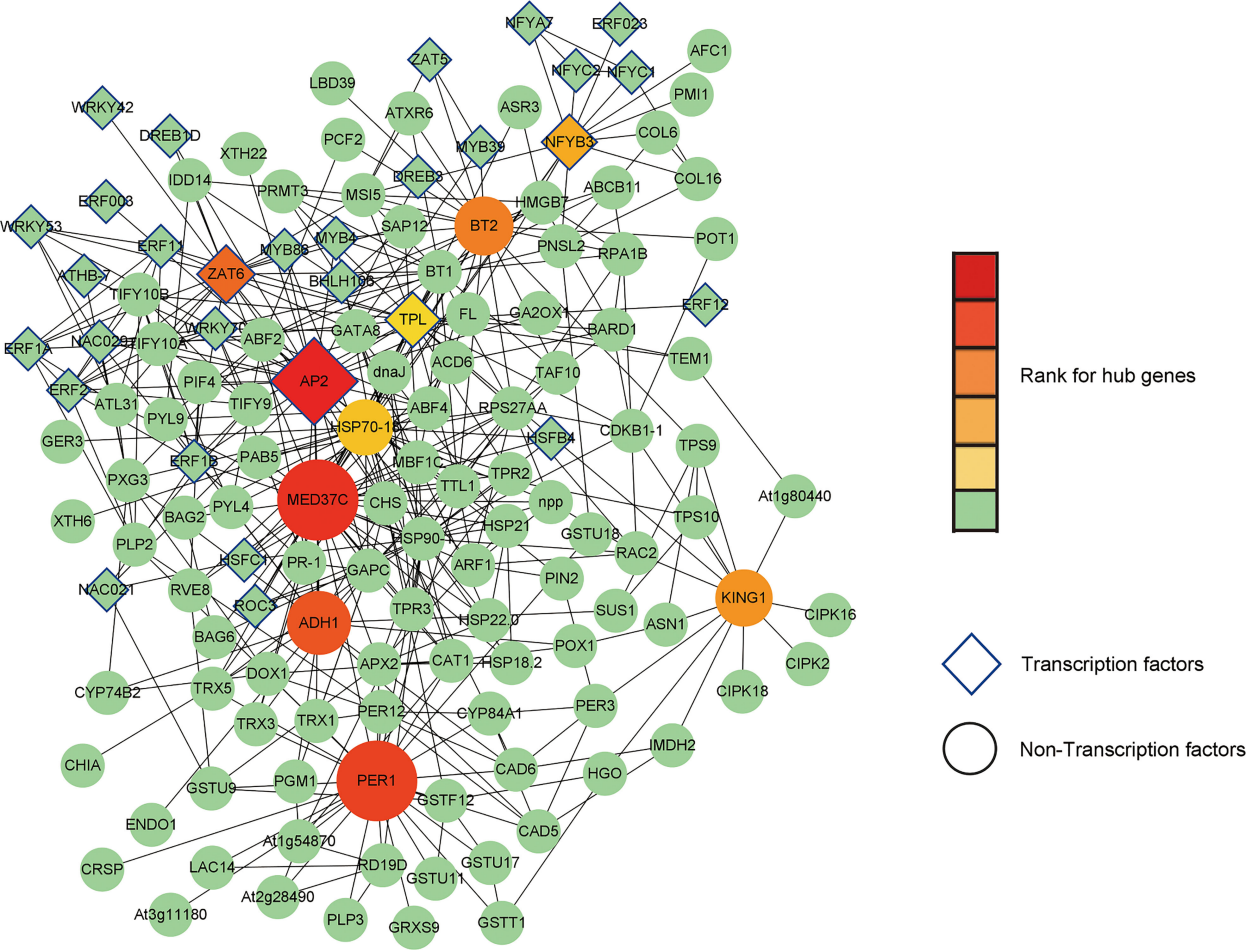
Ten putative hub genes and their interacting proteins. Transcription factors and non-Transcription factors are shaped as diamond and ellipse, respectively. Larger size and darker red color represent higher rank, while green color annotates lower rank.

Total 1039 DEGs at 0- 12 h (p< 0.05) were chosen as input data for prediction of their interaction in STRING database. Using Cytoscape, we input the output file of STRING search and obtained a network with 432 DEGs and their 1126 interacting events. We further ranked them and identified putative hub genes using cytohubba plugin (**Figure 12**).

From co-expression network, we identified ten putative hub genes including AP2 (TRINITY_DN33762_c0_g4), MED37C (TRINITY_DN32748_c0_g1), PER1 (TRINITY_N30585_c1_g4), ADH1 (TRINITY_DN34595_c0_g1), ZAT6 (TRINITY_DN29763_c0_g1), BT2 (TRINITY_DN28127_c0_g3), KING1 (TRINITY_DN32088_c0_g2), NF-YB3 (TRINITY_DN19275_c0_g1), HSP70-18 (TRINITY_DN21721_c1_g4), TPL (TRINITY_DN30465_c0_g4). Among them, TPL and NF-YB3 showed relatively higher log2FC values (7.76 and 2.98, respectively) in our RNA-seq data, suggesting that they play inductive and key roles in the response of lily bulbs to salt stress.

### Validation of transcriptome data through qRT-PCR

**Figure 13:**
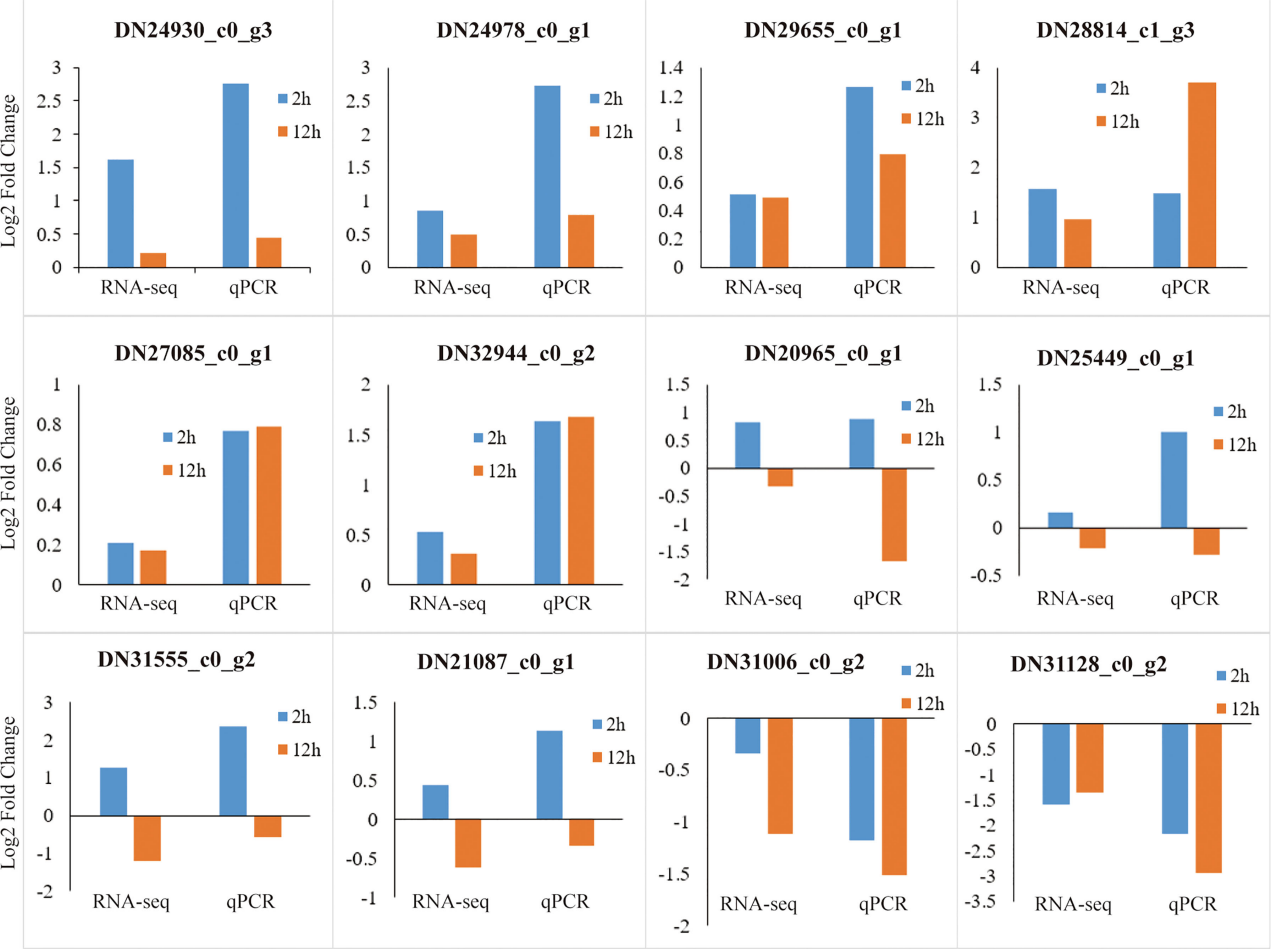
Verification of RNA-seq data through qRT-PCR. The abscissa represents comparison of the DEG expression level at 2 h (blue) and 12 h (red brown) to that at 0 h determined by RNA-seq and qRT-PCR, respectively. The ordinate represents the Log2 Fold Change values of the DEGs.

To verify accuracy and reliability of the RNA-seq data, we randomly selected twelve genes and further quantified their expression levels by qRT-PCR. The results showed that the expression patterns of the DEGs determined through qRT-PCR were highly consistent with the RNA-seq data (**Figure 13**).

## Discussion

Salt stress affects plant physiology mainly through ion toxicity, osmotic pressure and oxidative stress. Plants respond to these threats by regulating physiological and biochemical activities to adapt to or resist salt stress (Liang et al., 2018; Zhao et al., 2021). Among them, typical activities are accumulation of osmolyte compounds, ion-selective transmembrane transport and intracellular compartmentalization, ROS scavenging, and upregulation of salt tolerance genes including sensor genes, signaling genes and transcription factors (Shabala et al., 2015; Kumar et al., 2017; Liang et al., 2018; Zhao et al., 2021). Since the roots are the first organs to sense the salt stress (Duan et al., 2014; Karlova et al., 2021; Li et al., 2021), the research on salt stress have focused on the response of roots to salt stress, and then extends to other organs and tissues, mainly leaves and stems. The ornamental bulbous plants such as the lily have unique organs called the bulb that functions as nutrient or flowering organs. *L. pumilum* is considered to be a salt-tolerant ornamental plant, which may be related to the unique bulb organs function of *L. pumilum*. But it still lacks evidences showing that the bulb organs help plants overcome stressful environments such as salt stress. Therefore, we analyzed the *L. pumilum* bulb transcriptome data and identified the key genes regulating the bulb’s unique responses against salt stress at genome-wide scale.

The *L. pumilum* bulb transcriptome data at different time points (0, 2, or 12 h after salt stress treatment) were obtained by Illumina sequencing of total RNA samples, and analyzed. A total of 51566 unigenes were found and 1772 and 2324 DEGs were identified at initial (0-2 h) and later (2-12 h) periods, respectively. GO and KEGG enrichment analysis for these DEGs indicated that expression patterns of DEGs and KEGG annotated pathways showed slight difference between 0-2 h and 2-12 h. Among the DEGs, 958 DEGs were co-expressed during entire period (0-12 h) of salt stress, and their expression patterns were analyzed. It was found that many unigenes annotated as transcription factors were involved in the responses to salt stress. They belong to AP2/ERF, bHLH, bZIP, HD2/ZIP2, HSF, MYB, NAC, NF-YB and WRKY families. Among them, NF-YB3 transcription factor showed the most intense upregulation throughout entire period of salt stress (**Figure 10**). Beside the transcription factors, the unigenes annotated as metallothionein type 2 protein, vicilin like seed storage protein and bidirectional sugar transporter SWEET14 showed high expression levels and intense upregulation during entire period of salt stress.

**Figure 14:**
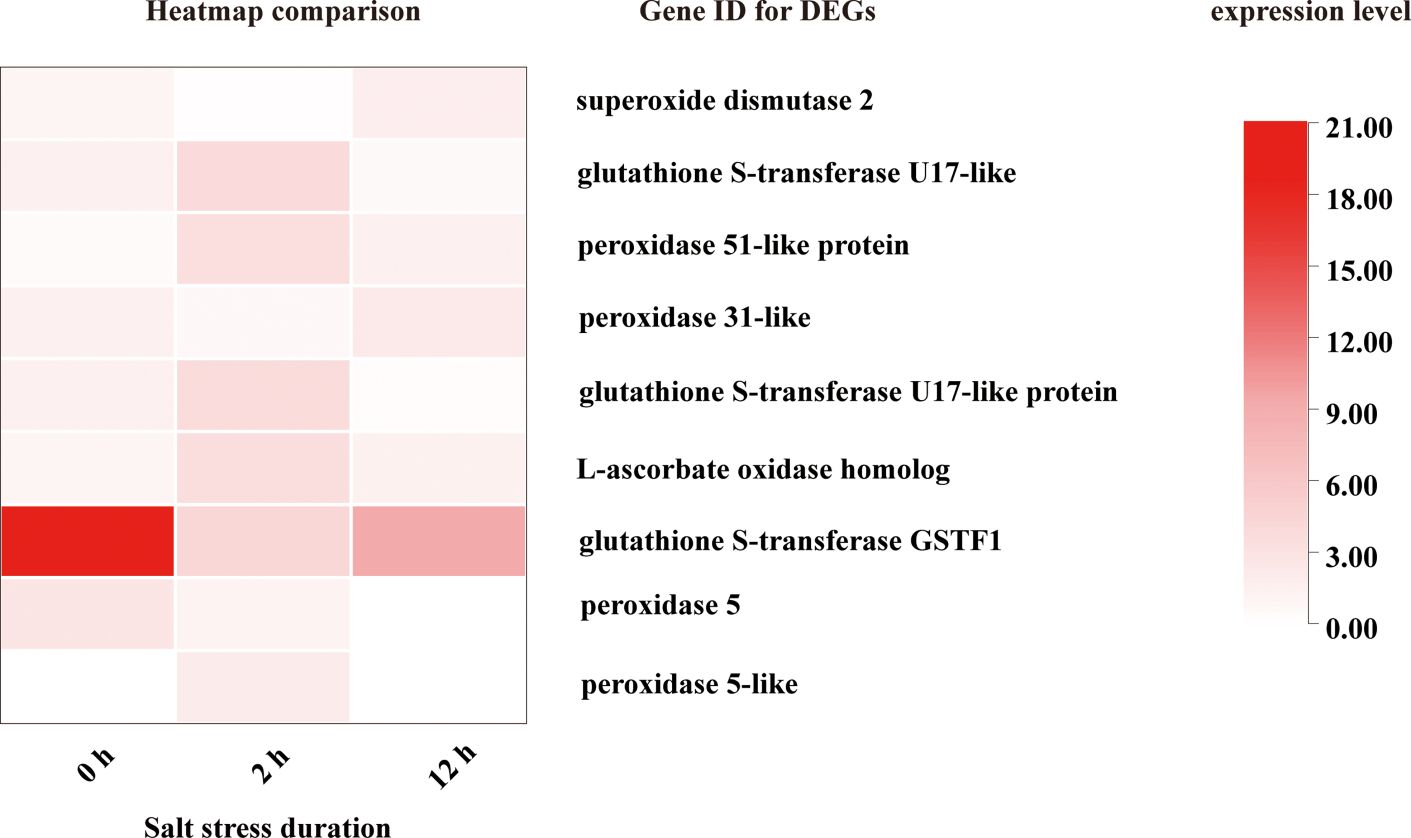
DEGs with ROS scavenging activity co-induced at both 0-2 h and 2-12 h.

From **Figures 8**, **9**, **11** it is obvious that stress response of the bulb organ was distinct from the typical responses such as ion-selective transmembrane transport and intracellular compartmentalization and ROS scavenging. For instance, in the transcriptome data of bulb organs, a number of genes responsible for responses to oxidative stress were identified as DEGs, 57 of which were predicted to be typical ROS scavengers, such as superoxide dismutase, peroxidase, and glutathione transferase, but most DEGs were downregulated. Some DEGs were upregulated, but their expression level was very low. In the 57 DEGs, 9 DEGs were co-expressed during entire period (both 0-2 h and 2-12 h) of salt stress and their expression patterns was shown in **Figure 14**. They also showed low expression levels and no intense upregulation. The effects of salt stress on bulb organs were different from those in roots, and bulb organs showed different responses, which may be related to the highly upregulated NF-YB3 transcription factor, metallothionein type 2 protein, vicilin like seed storage protein and bidirectional sugar transporter SWEET14. (**Figure 11**). NF-YB3 transcription factor is a subunit of the CCAAT-box binding factor family proteins (also called the Nuclear Factor Y). It is one of the large and ubiquitous transcription factors in all eukaryotic organisms including yeast, plant and animal, and plays important roles in flowering promotion, embryogenesis and seed maturation (Kumimoto et al., 2008; Laloum et al., 2013; Myers and III, 2018). NF-YB3 has also been reported to play a role in regulating plant responses to abiotic stresses such as drought, nutrient deficiency, salt, and temperature stress (Zhang et al., 2015; Yang et al., 2017; Sato et al., 2019; Dai et al., 2021; Zheng et al., 2021). In this study, we found that the expression of NF-YB3 transcription factor was upregulated in bulb organs under salt stress, suggesting that it was involved in regulation of salt stress. Co-expression network analysis also confirmed that *NF-YB3* acted as a hub gene during salt stress (**Figure 12**). *NF-YB3* is known to regulate not only the salt tolerance, but also the flowering time (Hou et al., 2014; Zhang et al., 2021). Thus, upregulation of *NF-YB3* in lily bulb organs upon salt stress can promote flowering of *L. pumilum*, but it requires further efforts to elucidate whether salt stress eventually promotes flowering in lily bulb.

Meanwhile, metallothionein type 2 protein, vicilin like seed storage protein were intensely upregulated. Metallothionein type 2 protein is a well-known metal ion binding protein that is characterized by high cysteine residue (Coyle et al., 2002). It plays significant roles in the detoxification and homeostasis of metal ions and ROS scavenging (Joshi et al., 2015; Jin et al., 2017; Babaei-Bondarti and Shahpiri, 2020), and these roles have already been demonstrated in *L. pumilum* (Wang et al., 2019). Metallothionein proteins are known to be regulated by several transcription factors such as the TFIID complex comprising TATA-binding protein associated factors, metal−responsive element (MRE)-binding transcription factor (MTF)-1, NF-Y family transcription factors (Takahashi, 2015). Vicilin like seed storage protein is a seed-specific protein that acts against biotic stress factors such as fungus and microbes (Vieira Bard et al., 2014; Jimenez Lopez, 2020; Ateeq et al., 2022; Bertonceli et al., 2022). Vicilin is also known to have the superoxide dismutase activity (Shikhi et al., 2018), which is able to resist against oxidative stress by scavenging ROS. Therefore, the “cavity” in anti-oxidation due to low activity of typical ROS scavengers can be filled by abundant metallothionein proteins and vicilin like seed storage proteins. The bidirectional sugar transporter SWEET14 was also the DEG with intense upregulation (**Figure 11**). SWEET protein was first discovered in plants as sugar transporters that mediate transmembrane sugar transport and is known to play diverse physiological roles in flower, fruit, seed development, gibberellin transportation, and apoplasmic phloem loading (Jeena et al., 2019; Ji et al., 2022). Sugars also can act as osmolytes that resist against osmotic imbalance during salt stress (Darko et al., 2019; Jeena et al., 2019) and upregulation of SWEET14 might consequently contribute to accumulation of sugars, thereby resisting osmotic damage from salt stress in this work.

Besides, E3-ubiquitin protein ligases were significantly upregulated during salt stress (**Figure 11**). E3-ubiquitin ligase mediated protein ubiquitination is one of the main post-translation modification pathways for protein which regulates various intracellular processes such as hormone signaling and stress resistance (Duplan and Rivas, 2014; Shu and Yang, 2017; Mandal et al., 2018; Kelley, 2018). The E3-ubiquitin ligases are known to regulate salt stress by participating in SOS pathway, MAPK Cascade, ABA signaling pathway, flowering pathway and ROS homeostasis (Wang et al., 2022). In our study, MAPK pathway was significantly activated during salt stress (**Figure 9**) and this might be associated with upregulation of E3-ubiquitin ligases. The enzymes also serve as central regulators of perception, regulation and biosynthesis of hormones such as auxin, brassinosteroid, cytokinin, ethylene, gibberellic acid, jasmonate, salicylic acid, and strigolactone (Kelley, 2018). In addition, PYL4 and protein phosphatase 2C are the major proteins in ABA signaling pathway; ABA inhibits PP2C through PYL4 (an ABA receptor protein), thereby releases SnRKs which then regulate energy homeostasis by promoting catabolism and repressing anabolism, thus actively inhibiting plant growth (Park et al., 2009; Zhang et al., 2020). Our study reported the downregulation of PYL4 and upregulation of protein phosphatase (**Figure 11**) which might lead to inactivation of ABA signaling, eventually relieving growth inhibition effect of ABA.

Bulb organs are different from roots that can directly sense and respond to salt stress, so the response of the bulb to salt stress would indirect and not exactly the same as the responses of root to salt stress. Substantiating this, our work provided the evidences that revealed bulb organ-unique responses against salt stress in *L. pumilum*, which are different from the previous results in roots or leaves. Altogether, lily bulb organs showed obvious salt tolerance that was evidenced by upregulation of non-typical ROS scavengers (such as the metallothionein type 2 protein) and osmolyte accumulation-related proteins (vicilin like seed storage protein, bidirectional sugar transporter SWEET14). Besides, phytohormone signaling factors (E3-ubiquitin protein ligases and protein phosphatase 2C) were upregulated, suggesting that salt tolerance of the lily plants requires hormone signaling. Among the DEG transcription factors including DREB, ERF, bHLH, bZIP, HD2/ZIP2, HSF, MYB, NAC, NF-YB, and WRKY (**Figure 10**), NF-YB3 was the only factor that showed full period upregulation. Co-expression analysis further confirmed that NF-YB3 is a putative hub factor that regulates lily bulb response against salt stress. NF-YB3 is a well-known transcription factor that promotes flowering in plants and Lily bulb is a reproductive organ of *L. pumilum*. Therefore, upregulation of NF-YB3 in the bulbs under salt stress can promote flowering of lily. Further researches are still required to unveil which upstream signaling factors induce metallothionein type 2 protein, vicilin like seed storage protein, bidirectional sugar transporter SWEET14, E3-ubiquitin protein ligases and protein phosphatase 2C, as well as which downstream genes of NF-YB3 transcription factor are induced during salt stress to govern NF-YB3-mediated salt stress responses or flowering signals in *L. pumilum*.

## Conclusion

As a wildflower plant with high salt-alkali tolerance, *L. pumilum* is regarded as an important germplasm resource for the lily stress resistance breeding in achieving efficient gardening. The bulbs are the important organs for lily propagation and other uses; flowers are developed from them and more importantly, they can be used for culinary and medicinal purposes. Besides, these unique bulb organs are believed to play significant roles in regulating high salt-alkali tolerance of *L. pumilum*, albeit lacking evidences.

Our work for the first time revealed that bulb organs of *L. pumilum* respond significantly against salt stress, which is different from root organs. Through transcriptome analysis, we identified DEGs associated with salt stress and then screened several key genes with intense upregulation degree. They include NF-YB3 transcription factor, metallothionein type 2 protein, vicilin like seed storage protein and bidirectional sugar transporter SWEET14. It has been commonly accepted that stress responses mainly lead to increase in ROS level and always include upregulation of typical ROS scavengers like superoxide dismutase, peroxidase, and glutathione transferase, however, they were not differentially expressed and rather the non-typical ROS scavengers such as the metallothionein type 2 protein, and vicilin like seed storage protein were upregulated in our work. Imbalance in osmotic pressure caused by salt stress is another type of damage and plants normally respond with the accumulation of osmolytes such as sucrose. Upregulation of the bidirectional sugar transporter SWEET14 protein reported in this study may promote the sucrose accumulation favorable for maintaining osmotic homeostasis under salt stress. We also reported that the hormone signaling proteins such as E3-ubiquitin protein ligases, PYL4 and protein phosphatase 2C were upregulated, suggesting the role of hormones in the bulb organ responses to salt stress. Besides, co-expression analysis of the DEGs confirmed that NF-YB3 transcription factor acted as a hub gene. Overall, these results lay a foundation for further research on the response mechanism of bulb to salt stress in *L. pumilum* under salt stress, which has important reference value for studying the molecular mechanism of regulation network of lily coping with different abiotic environmental stress, and provide key genes information for tolerance breeding of lily.

## Additional file

qRT-PCR Primer information was shown in Additional file.

## Data availability statement

The datasets presented in this study can be found in online repositories. The names of the repository/repositories and accession number(s) can be found below: https://www.ncbi.nlm.nih.gov/, PRJNA851552.

## Author contributions

KS, UP, and SS conceived and designed the experiments. KS and SS conducted the experiments. KS, UP, YW, and HY elaborated and analyzed experimental data, YZ, KS, and UP wrote the manuscript. All authors contributed to the article and approved the submitted version.

## Funding

This work was supported by the Fundamental Research Funds for the Central Universities [grant numbers 2572019BK04]; and the Natural Science Foundation of Heilongjiang Province [grant numbers LH2019C004].

## Conflict of interest

The authors declare that the research was conducted in the absence of any commercial or financial relationships that could be construed as a potential conflict of interest.

## Publisher’s note

All claims expressed in this article are solely those of the authors and do not necessarily represent those of their affiliated organizations, or those of the publisher, the editors and the reviewers. Any product that may be evaluated in this article, or claim that may be made by its manufacturer, is not guaranteed or endorsed by the publisher.
